# Effectiveness of Additive Manufactured Titanium Implants in the Reconstruction of Large Cranial Defects: Case Series and Review of Literature

**DOI:** 10.1007/s12663-023-02085-1

**Published:** 2023-12-28

**Authors:** Ashish Chakranarayan, Pushpa Kumari, Shakil Ahmad Nagori, Manoharan Dwark Sudhan, P. Suresh Menon, Anita Kapri

**Affiliations:** 1 University of Central Lancashire, Preston, Lancashire, PR1 2HE UK; 2 INHS Kalyani, Visakhapatnam, AP 530005 India; 3 INDC Danteshwari, RC Church Colaba, Mumbai, MH 400005 India; 4 Neurosurgery Centre, INHS Asvini, Mumbai, India; 5 Department of Oral & Maxillofacial Surgery, Vydehi Institute of Dental Sciences & Research Center, Whitefield, Vijayanagar, Nallurhalli, Bengaluru, Karnataka 560066 India

**Keywords:** Craniectomy, Craniotomy, Cranioplasty, Residual cranial deformity, Additive manufacturing, 3D printing, Cranial bone, Titanium cranial implant

## Abstract

**Introduction:**

Replacement of lost soft and hard tissues of the human body has always been a daunting task across all surgical specialties. Reconstruction of a cranial deformity is challenging due to the functional and cosmetic requirements. A major constraint with large cranial bony deformity reconstruction is the nonavailability of graft of a specific shape and size.

**Materials and Method:**

A total of four cases of large cranial defects which included three cases of unilateral and one case of midline residual deformity were reconstructed at our center using customized titanium implants. These implants were fabricated using additive manufacturing/3D printing technology utilizing computerized tomographic data.

**Conclusion:**

The additively manufactured titanium implants appear to be a viable option in the reconstruction of large cranial defects.

## Introduction

As far as the human body is concerned, “God given” is peerless. In the pursuit of immortality, clinicians and scientists have been persevering to find ideal replacements for diseased or lost human tissue. Due to various reasons, the replacement of missing soft and hard tissues of the human body has always been a daunting task across all surgical specialties. The ideal replacement material would be a similar tissue or organ, and in a large number of cases, owing to bilateral similarity, native tissue such as skin, mucosa, muscle, bone, organ, etc., is available for harvest. However, the size, shape, and vascularity are often the important limiting factors. Residual deformity of the cranium following trauma or surgery is commonly encountered in clinical practice [1]. Reconstruction of a cranial deformity is peculiar due to the functional and cosmetic requirements. Historically, based on the available evidence, the Incan civilization was probably performing precious metal cranioplasty-like procedures; however, the first documented report is by Fallopius in the sixteenth century [2]. The benefits of cranial reconstruction in allaying the symptoms of the syndrome of the trephine/sinking flap syndrome (SFS) have been widely reported in the literature thereafter [3]. In many cases the neurosurgeon can perform an access osteotomy which largely preserves the shape and size of the bone flap. These flaps are then either stored in the abdominal wall or anterior thigh for later use. However, in many instances, the access procedure leaves the bone flap unusable, thereby warranting a reconstruction using other materials. This is done to provide protection to the underlying brain tissue and to restore cosmetics. A major constraint with large cranial bony deformity reconstruction is the nonavailability of graft of a specific shape and size. Over the years, various methods have been developed and used to replace the missing cranial bone to reconstruct a residual deformity of the skull [2]. The most preferred option is a local bone graft harvested from the contralateral side of the cranium [4]. Some of the other methods which have been popular are the use of polymethyl methacrylate (PMMA) plates, porous polyetheretherketone (PEEK), and titanium meshes. Though being relatively cost-effective and commonly available, all these methods have severe limitations in terms of allergic reaction, percutaneous exposure, and inability to restore desired shape, respectively [5]. The use of cranial distraction osteogenesis has been widely reported for management of different kinds of synostotic conditions; however, the employability of this technique for treating residual deformity has not been observed much [6]. Technological advancement in the field of rapid prototyping has made available to the clinician, a very viable substitute for the lost cranial bone tissue, which may be able to overcome most limitations of the conventional methods of cranial deformity reconstruction. Our study attempts to elaborate the use of customized titanium implants which are commonly referred to as patient-specific implants (PSI). These were fabricated using the additive manufacturing technology. This recently emerged technology has shown exponential progress in the fields of medicine, aerospace, energy, consumer products, and transport [7]. Using the STROBE guidelines to the best possible extent, we intend to demonstrate the clinical effectiveness of these implants as a viable substitute to the other methods of cranial reconstruction and review the relevant literature with regard to the reconstruction techniques and their advantages and disadvantages.

## Materials and Method

**Fig. 1:**
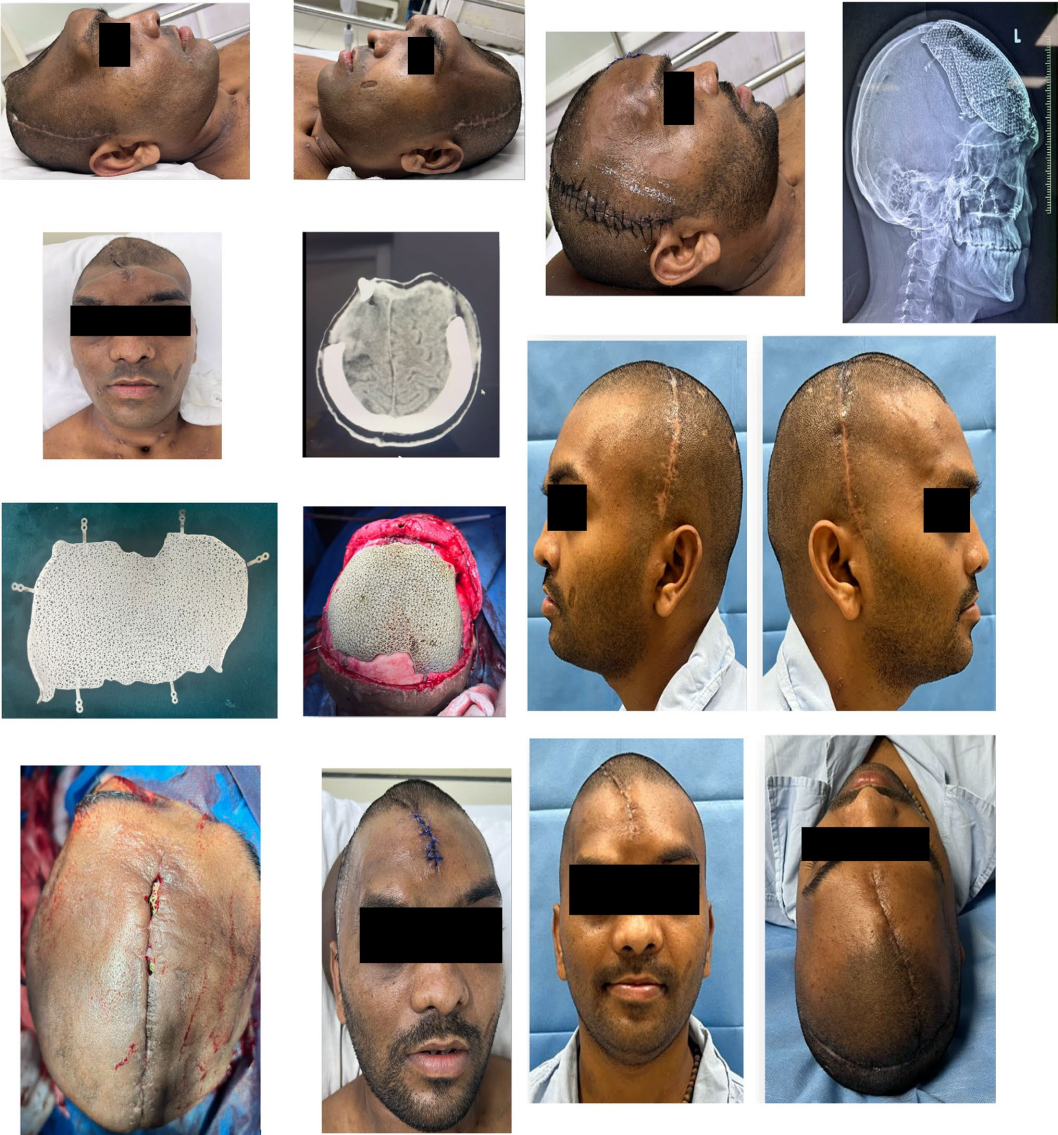
Reconstruction of large bilateral cranial defect using additively manufactured titanium implant

**Fig. 2:**
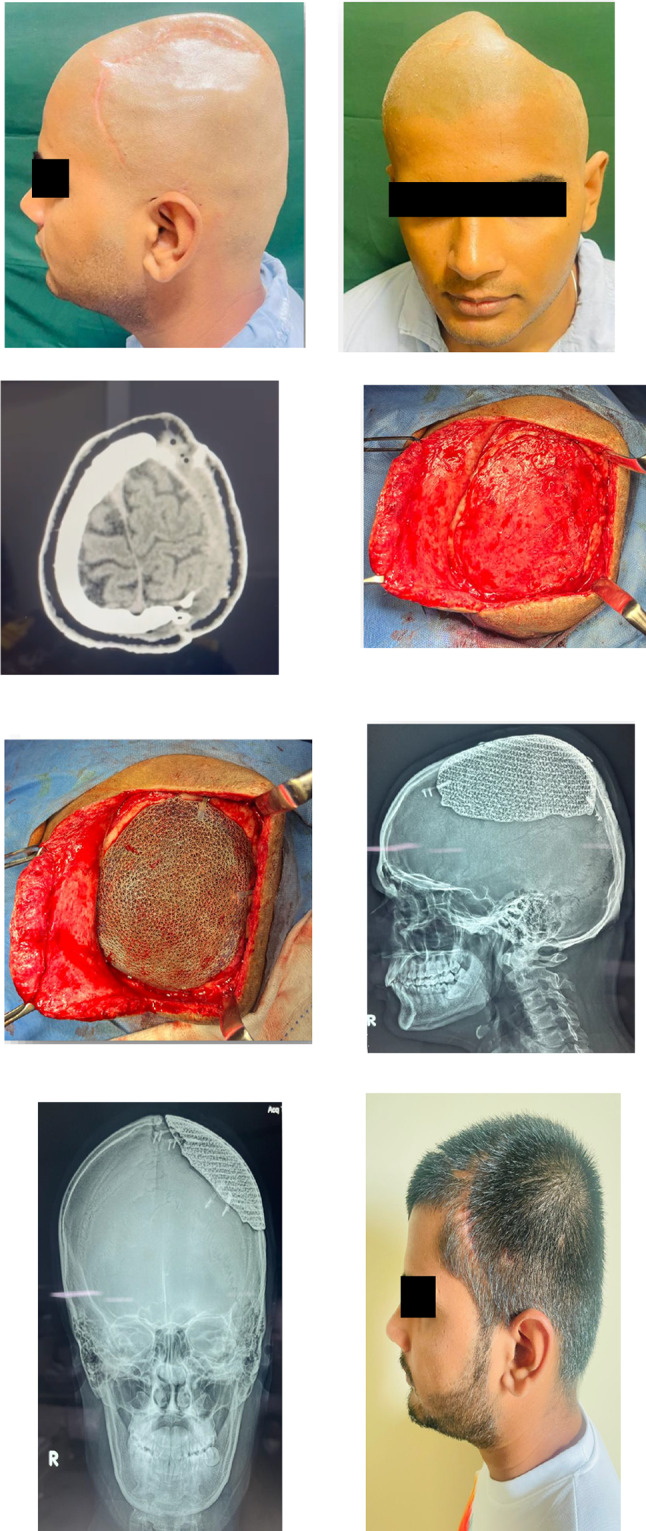
Reconstruction of large unilateral cranial defect using additively manufactured titanium implant

**Fig. 3:**
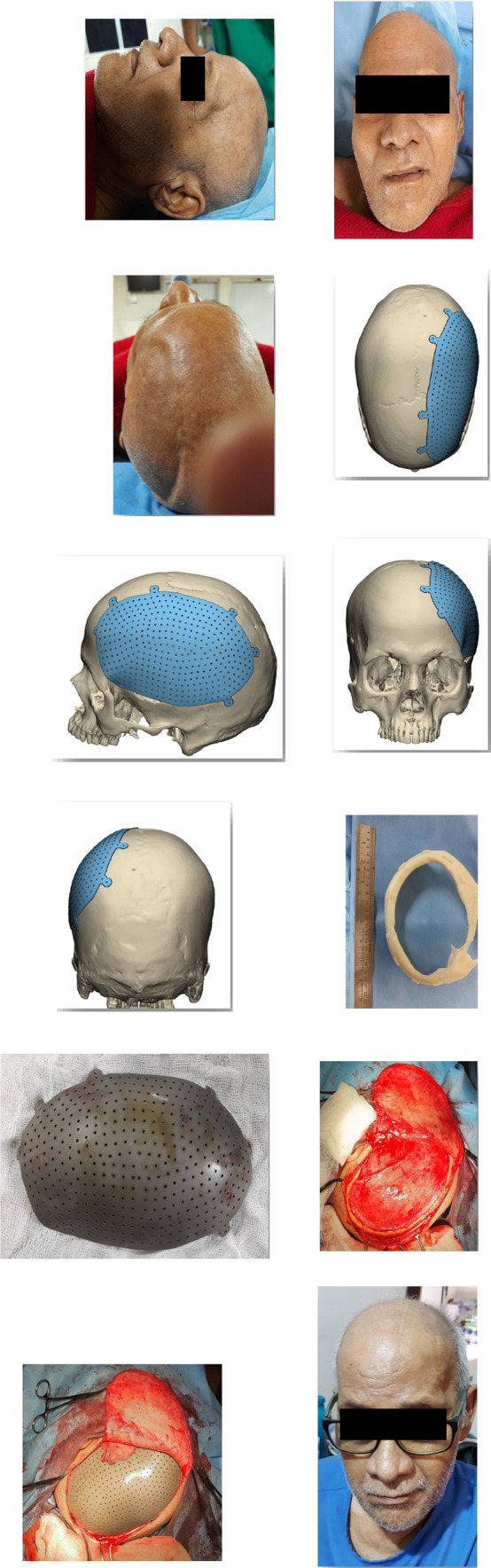
Designing and steps in the construction and placement of large additively manufactured titanium cranial implant

The retrospective descriptive study included cases operated at our center from September 2020 to April 2022. All cases were referred from the Department of Neurosurgery for reconstruction of cranial residual deformity. The surgery protocol was approved by the institutional review board and the patient’s consent was obtained for administration of general anesthesia, surgical procedure, and scientific publication subsequently. The age range was from 28 to 65 years and comprised of all male patients. The Glasgow Coma Scale (GCS) in two cases was 15 and in one case 13 and 8 each [Table 1]. One case was secondary to improper storage of the bone flap after a decompressive craniectomy in a case of uncontrolled hypertension [Fig. 3]. In this case, although the shape and size of the bone flap were optimal, it was deemed unfit for replacement due to improper storage after the primary surgery. The remainder of three cases were of residual deformity after a neurosurgical intervention for management of head injury due to road traffic accidents [Table 1]. All cases were systemically stable with no uncontrolled comorbidities. Routine preoperative blood and urine examination was done as per the anesthesiology requirement. Computerized tomography (CT) was done in 1-mm axial and coronal slices. Volume-rendered radiographic images were developed based on these data. The CT data were provided to the manufacturer on a compact disc for fabrication of titanium PSI for all cases [Table 1]. The surgery was carried out under general anesthesia with a reinforced oral endotracheal tube. Preoperatively, injection dexamethasone 8 mg iv was administered approximately 1 h prior to the procedure along with a broad-spectrum antibiotic cover which included injection amoxicillin 1000mg + clavulanic acid 200mg iv, injection amikacin 750 mg iv, and injection metronidazole 500 mg iv. The perioperative anesthetic drugs and management were as per the standard protocols. The patient was scrubbed neck above and draped aseptically. The operating table was adjusted to a comfortable head-up position. The incision line which was along the scar of the previous surgical intervention was infiltrated with 10 to 15 cc of 1:100,000 adrenaline in normal saline at the supra-pericranial level. A flap was raised to delineate the scalp from the dura mater. In all unilateral cases, the elevation of the flap to expose the area of interest was uneventful [Figs. 2, 3]; however, in the patient requiring bilateral reconstruction, there were two breaches in the underlying pericranium/dura layer [Fig. 1]. These were uneventfully sutured using 3–0 resorbable suture. Sufficient exposure of the area of interest involved exposure of the bony margins of the defect in anterior, superior (midline), and posterior edges of the defect; however, the inferior edge which was below or at the level of the zygomatic arch was left unexposed. Pericranium was incised to expose the underlying bone at the fixation sites and along the bony edges of the defect to ensure optimal seating of the implant. The fit of the autoclaved implant was verified, and the same was fixed using 2-mm-diameter x 7-mm-long titanium screws. Hemostasis was verified, and the scalp flap was replaced after insertion of a suction drain between the implant and the flap, which was partially activated to avoid undue suction on the surrounding structures and was removed on the second postoperative day. The flap was replaced using 3–0 resorbable sutures in the deeper layer and stainless-steel staples on the skin. Pressure dressing was applied over the incision line using Elastoplast [Figs. 1,2,3]. Postoperatively, the patient was maintained nil per os for 6 h, and injection paracetamol 1 g iv was used for postoperative pain control along with injection dexamethasone 8 mg, which were replaced by tablet ibuprofen 400mg + paracetamol 500 mg combination after the second postoperative day. The patients were under the same antibiotic cover for 3 to 5 days and were discharged after 5 to 7 days postoperatively. The skin staples were removed after 10 days. All cases were reviewed after a month.

**Table 1:** Patient demographics

S. no.	Etiology	Number of cases	Number of sites	GCS		Number and type of neurosurgical surgeries/procedures prior to cranioplasty	Time duration after the last neurosurgical intervention (months)
				Pre-op	Post-op		
1	RTA	1	Bilateral	15	15	03/Craniectomy/brain abscess drainage	02
2	RTA	1	Unilateral	15	15	01/Craniectomy	02
3	Stroke	1	Unilateral	13	15	01/Craniectomy	24
4	RTA	1	Unilateral	8	8	02/Craniectomy/VP shunt placement	02

The laboratory protocol for construction of the PSI involved the use of CT data in Digital Imaging and Communication in the Field of Medicine (DICOM) protocol, which was used to construct a 3D model in Standard Tessellation Language (STL) file. This was used to design the implant using mirroring and merging using computer software to obtain a bilaterally symmetrical shape and size. The design thus developed was used in a machine employing electron beam melting (EBM)/selective laser melting (SLM) technology to construct the implant in a cyclic process involving preheating the powdered titanium alloy, which was then scanned and melted to develop a solidified layer. Additional layers were repeatedly added till the desired form was achieved.

## Results

A total of four cases were operated for reconstruction of residual deformity of the cranium using additively manufactured titanium PSI with the intention of providing protection to the underlying brain tissue, improve brain physiology, and cosmetics. The cases secondary to RTA were younger in age as compared to the one after a stroke. Relevant patient demographics are as per Table 1. All cases demonstrated a significant improvement in cosmetics [Figs. 1, 2, 3]. The two cases which were operated with GCS 15 showed no change in the GCS postoperatively; however, on review, the patients reported a sense of well-being and improved confidence. The patient who was on GCS 13 preoperatively demonstrated a significant improvement in cognition postoperatively. However, the case which was operated at GCS 8 continues to be at the same level of consciousness and has shown no signs of improvement till date. No postoperative complaints at the surgical site and/or otherwise have been reported so far.

## Discussion

A decompressive craniectomy is known to be lifesaving in the event of a refractory intracranial hypertension [8]. This could happen due to a variety of reasons and is broadly defined as a raised intracranial pressure (ICP) beyond 20 to 22 mm Hg for a sustained period of 10 to 15 min [9]. Traumatic brain injury, vascular pathology, tumors, etc., are the common etiopathological reasons for a raised ICP. Contemporarily, large decompressive craniectomies, that is, the ones involving a complete hemisphere/frontotemoproparietal and the bilateral/bifrontal procedures along with an augmentative duroplasty, are known to produce a clinically acceptable outcome [9]. As a common practice, the osteotomized bone flap is stored in the abdominal wall or anterior thigh, and the patient is observed for a prolonged period prior to a cranioplasty. It is recommended to undertake an early reconstruction of the cranium to mitigate the symptoms of the sinking flap syndrome which is known to clinically present as altered consciousness and cognition levels, speech deficit, psychosomatic disturbances, and seizures. Altered cerebral metabolism, changed pattern of CSF, cerebral blood flow, and atmospheric pressure have been suggested as the possible pathophysiologies of this syndrome [10]. To avoid an additional surgery (cranioplasty) and allay the side effects of trephination, Claudia et al. proposed hinging the bone flap prior to closure of the craniectomy (the Tucci flap). A similar technique known as the “In situ hinge craniectomy" was advocated by Kathryn et al. with reasonable success [11]. Unfortunately, in many cases, the bone flap is unviable to be replaced back during a cranioplasty, thereby giving rise to the need of finding a substitute.

Intriguingly, craniectomy which was earlier designated as trephination finds much more mention in the literature as compared to cranioplasty, and there seem to be large gaps in the recorded history in this regard. After the sixteenth-century gold plate cranioplasty reference, the next one is of a xenograft using a dog bone in a Russian man’s skull in 1680. Cranioplasty gained popularity in the twentieth century, and various techniques have been advocated thereafter. A variety of materials ranging from autogenous bone, xenografts, metals, and nonmetallic substances have been reported for cranial reconstruction. The common ones are cranium, sternum, scapula, fibula, tibia, rib, fat, fascia, canine bone, gold, silver, titanium, aluminum, lead, vitallium, tantalum, ticonium, stainless steel, hydroxyapatite, PMMA, and PEEK [2].

**Fig. 4:**
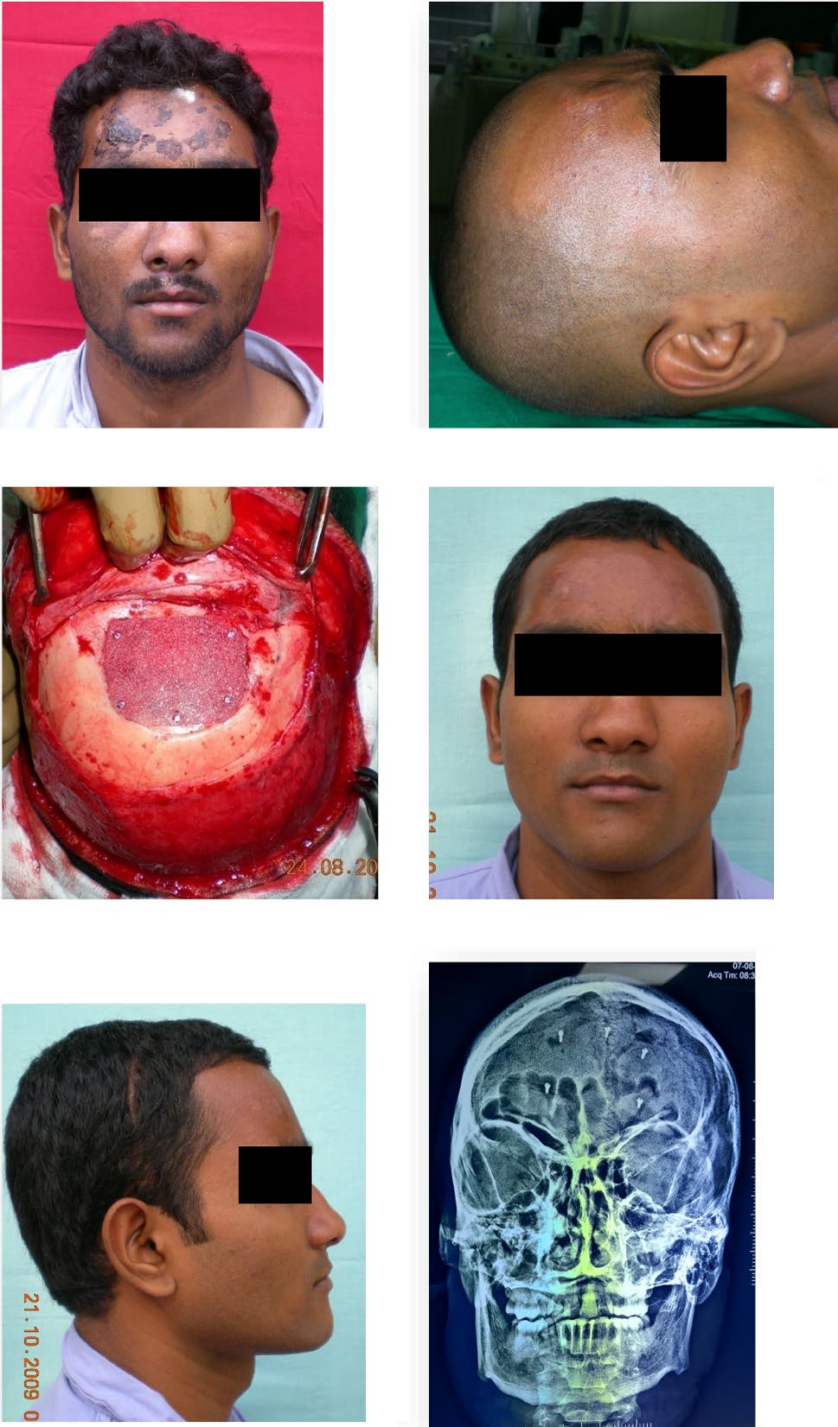
Use of porous alloplastic material as onlay graft for cranial reconstruction

**Fig. 5:**
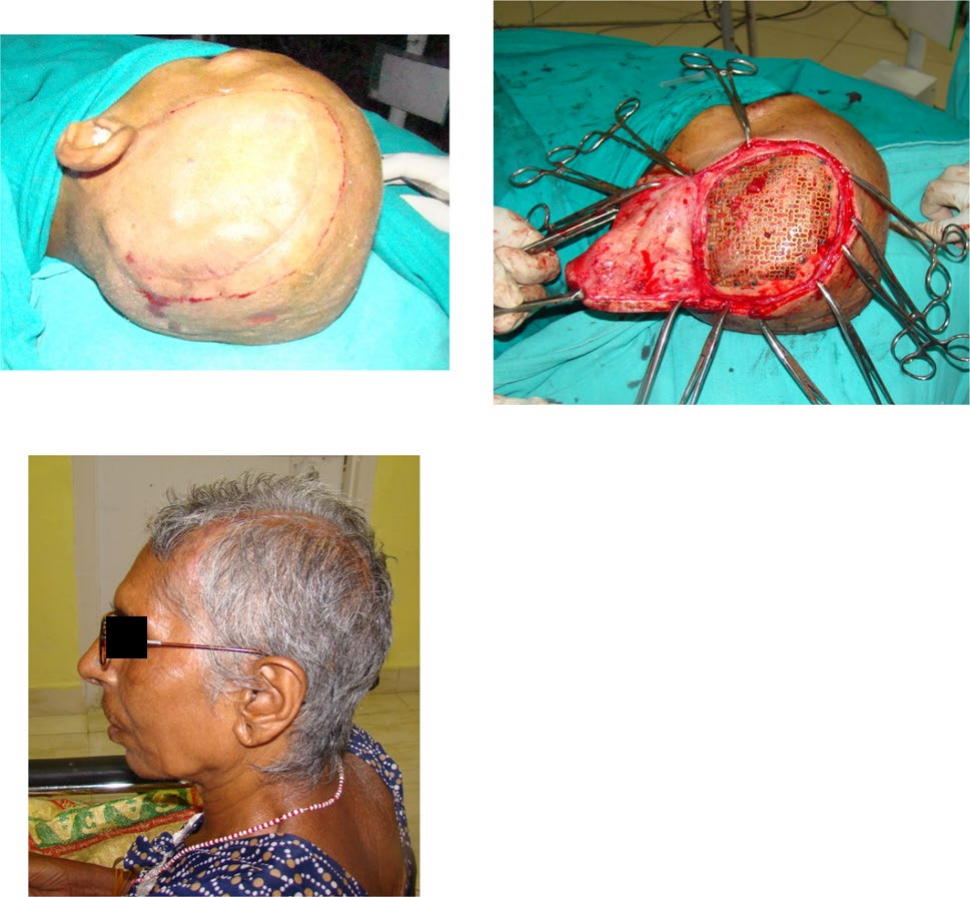
Use of pre-formed titanium mesh for cranial reconstruction

**Fig. 6:**
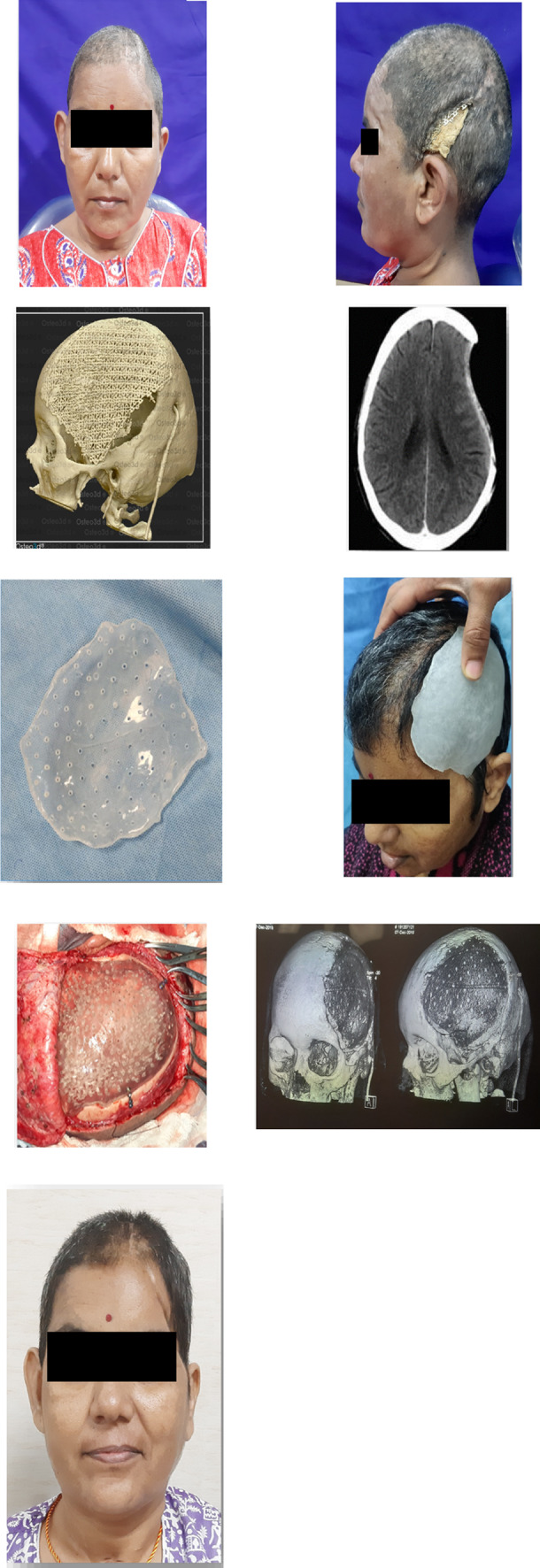
Percutaneous perforation and exposure of pre-formed titanium mesh used for cranial reconstruction

In cases where the defect is small and does not require a very specific shape, native bone harvested from the contralateral side of the cranium is a good source of bone [4], which could be used to bridge the residual deformity. However, with this technique the volume of available bone is limited and requires surgical expertise to harvest the graft [12]. Initially reported in the 1940s, PMMA plates used to be the material of choice for a long time, for reconstruction of larger defects, as they are easy to fabricate and are cost-effective [13] (Fig. 6). However, allergic reaction due to the leaching of the monomer from the PMMA, fracture of the plate, and cumbersome retrievability in case of a subsequent injury were reported as the limiting factors in its use [14]. Sane et al. have reported titanium mesh-reinforced prosthesis to overcome the strength, fragility, and retrievability drawbacks associated with a PMMA prosthesis [15]. Other porous materials like polyethylene, PEEK, and hydroxyapatite have also been reported in cranial reconstruction, but owing to their strength characteristics, these seem to be better suited to be used as onlay grafts for cranial recontouring [16] [Fig. 4]. Titanium mesh, which is available in various sizes, has been reported to be a much better alternative to the PMMA plates as far as the infection rates are concerned. However, the major limitation of these meshes is their inability to be bent into specific shapes, which limits their use to small-/medium-sized defects (Fig. 5). Additionally, there have been reports of skin erosion and implant exposure with the use of these meshes [17] (Fig. 6).

**Fig. 7:**
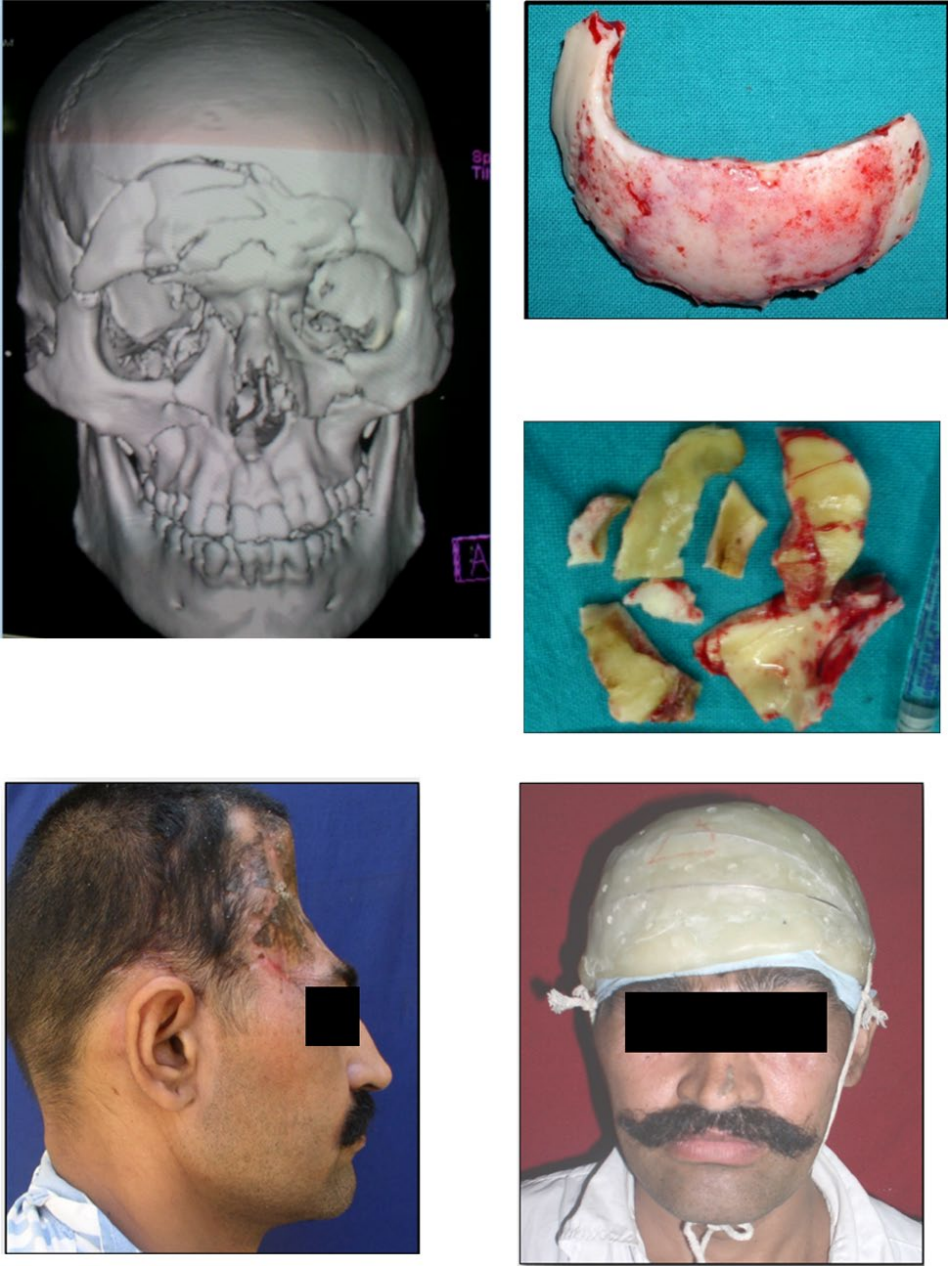
Use of protective custom-made acrylic helmet after craniectomy

The reconstruction of a large residual deformity of the cranium is known to be technically challenging and expensive. Some centers advocate the use of a customized helmet to delay or defer a cranioplasty [18]. In our opinion, such practices are of historical value [Fig. 7]. The additive manufacturing technology seems to provide an option for manufacturing cranial implants to cover all kinds of defects. The main advantage of this technology is its capability to deliver customized large implants of a specific shape without compromising the strength, precision, and biocompatibility [19]. Such reconstructions are not viable with conventional bone or polymeric material, as they lack optimal volume and mechanical properties, respectively. Additive manufacturing is a method of building an object in a layer-wise manner and is commonly referred to as 3D printing. The technology dates to the 1980s where it was used to create prototypes which were usually not functional and was known as rapid prototyping. Overtime, it has evolved into an efficient method of manufacturing complex working devices. The process involves developing a design of the object using the computer-aided designing (CAD) technology, which is then used to print the same by the computer-aided manufacturing (CAM), using a 3D printer [20]. In the manufacturing of cranial hard tissue implants, DICOM data from a CT scan are used to generate a design of the implant, which is then used to fabricate the same using the electron beam melting (EBM)/selective laser melting (SLM) of powdered titanium alloy which involves laser-assisted melting of the powdered alloy and solidification in the desired shape [21, 22]. Additive manufacturing technology has been used to create cranial implants with other materials as well, like PEEK, PMMA, and hydroxyapatite [23–25]. However, in our study, titanium was used for the management of these cases based on its proven superior mechanical and biocompatible properties. Introduced in the 1960s, the medical grade/grade 5 alloy (Ti6Al4V) [26] is the contemporary metal of choice for manufacturing of hard tissue implants because of ease of fabricability, biocompatibility, etc. This quality enables the manufacturing of complex shapes which mirrors the contralateral/uninvolved side, thereby creating an implant which mimics the presurgical dimensions. This property is particularly valuable in creating implants for defects crossing the midline, as these cannot be optimally managed using other kinds of materials. Postmanufacturing these implants are convenient to handle. These can be autoclaved routinely prior to placement on the patient and fixed with commonly available stock titanium screws. In our experience, the ease of placement, precision fit of the implant, and cosmetic outcome were clinically satisfactory. Though postoperative limitations of metal artifacts in case of subsequent diagnostic evaluation and complications such as pain at the surgical site, infection, exposure due to overlying skin erosion, displacement of implant due to failure of fixation, and wound dehiscence have been reported [22], majority of patients in our study reported improvement in general well-being after the procedure, and no clinically significant complications have been reported so far.

## Conclusion

In our experience, the additively manufactured titanium implants for cranioplasty are a viable option for reconstruction of large residual deformity cases, especially the bilateral defects. The advantages outweigh the only perceivable disadvantage of not being economical as on date. With the improvement in this technology, the manufacturing cost is likely to become more affordable in the times to come, and more extensive studies may improve the understanding and utilization of this technology.
